# Disruption of *Tmem30a* results in cerebellar ataxia and degeneration of Purkinje cells

**DOI:** 10.1038/s41419-018-0938-6

**Published:** 2018-09-05

**Authors:** Yeming Yang, Kuanxiang Sun, Wenjing Liu, Lin Zhang, Kun Peng, Shanshan Zhang, Shujin Li, Mu Yang, Zhilin Jiang, Fang Lu, Xianjun Zhu

**Affiliations:** 1 Sichuan Provincial Key Laboratory for Human Disease Gene Study, Sichuan Provincial People’s Hospital, University of Electronic Science and Technology of China, Chengdu, Sichuan China; 2 Chengdu Institute of Biology, Sichuan Translational Medicine Research Hospital, Chinese Academy of Sciences, Chengdu, China; 3 Department of Laboratory Medicine, Sichuan Academy of Medical Sciences and Sichuan Provincial People’s Hospital, Chengdu, Sichuan China; 4 Institute of Laboratory Animal Sciences, Sichuan Academy of Medical Sciences and Sichuan Provincial People’s Hospital, Chengdu, Sichuan China

## Abstract

Phospholipids are asymmetrically distributed across mammalian plasma membrane with phosphatidylserine (PS) and phosphatidylethanolamine concentrated in the cytoplasmic leaflet of the membrane bilayer. This asymmetric distribution is dependent on a group of P4-ATPases named PS flippases. The proper transport and function of PS flippases require a β-subunit transmembrane protein 30 A (TMEM30A). Disruption of PS flippases led to several human diseases. However, the roles of *TMEM30A* in the central nervous system remain elusive. To investigate the role of *Tmem30a* in the cerebellum, we developed a *Tmem30a* Purkinje cell (PC)-specific knockout (KO) mouse model. The *Tmem30a* KO mice displayed early-onset ataxia and progressive PC death. Deficiency in *Tmem30a* led to an increased expression of Glial fibrillary acidic protein and astrogliosis in regions with PC loss. Elevated C/EBP homologous protein and BiP expression levels indicated the presence of endoplasmic reticulum stress in the PCs prior to visible cell loss. Terminal deoxynucleotidyl transferase dUTP nick end labeling (*TUNEL*) analysis suggested that apoptotic cell death occurred in the cerebellum. Our data demonstrate that loss of *Tmem30a* in PCs results in protein folding and transport defects, a substantial decrease in dendritic spine density, increased astrogliosis and PC death. Taken together, our data demonstrate an essential role of *Tmem30a* in the cerebellum PCs.

## Introduction

Phospholipids are asymmetrically and dynamically distributed across plasma membranes^1,2^. In mammalian plasma membranes, phosphatidylserine (PS) and phosphatidylethanolamine are concentrated in the cytoplasmic leaflet of the membrane bilayer, whereas phosphatidylcholine and sphingolipids almost exclusively localized to the exoplasmic leaflet^3^. Mounting evidence indicates that the asymmetric distribution of lipids is largely determined by P4-ATPases, a diverse group of lipid transporters that use the energy of ATP hydrolysis to move specific lipids from the outer to the inner leaflet of membrane bilayer^4–7^. This process is termed “lipid flipping”, accordingly P4-ATPases are also known as flippase. P4-ATPases generate and maintain the phospholipids asymmetry, which plays crucial roles in membrane stability, vesicle trafficking, blood coagulation, cell polarity and migration, clearance of apoptotic cells, cell division, sperm capacitation, and cell signaling^8–13^.

Notably, P4-ATPases are essential components of the Golgi, plasma membrane and endosomal system that play critical roles in vesicle formation and trafficking^14^. Previous studies have reported that loss of Drs2 flippase activity at the trans-Golgi network (TGN) in yeast blocks the initiation of clathrin-coated vesicle biogenesis^9,15^. Drs2 is also essential for bidirectional vesicular transport between the TGN and early endosomes^9^. In *Arabidopsis thaliana*, *ALA3* is required for the formation of post-Golgi vesicles at the plant root tip in actively secreting cells^16^. Human *ATP8B1* participates in apical protein localization^17,18^. P4-ATPases may also participate in protein glycosylation in the endoplasmic reticulum (ER), which is an essential activity for the synthesis, folding, and function of glycoproteins in eukaryotes^19^. Collectively, these data support the hypothesis that P4-ATPases play a fundamental role in vesicle-mediated protein transport in the Golgi and endosomal systems.

In mammals, 14 P4-ATPases are encoded in the genome. Thus far, several P4-ATPases have been associated with severe human disorders^20^. For instance, mutations in *ATP8B1* cause progressive familial cholestasis, a disease associated with defects in bile secretion^21^. Besides, emerging evidence suggests that P4-ATPases play important roles in the central nervous system and in neuronal survival. Mutations in *Atp8a2* cause axonal dystrophy, axonal degeneration in the medial cerebellar nuclei, and retinal degeneration in mice, and a severe neurological disorder in humans that is characterized by cerebellar ataxia, mental retardation, and disequilibrium syndrome^22–25^. In vitro data obtained using neuronal PC12 cells and rat hippocampal neurons also indicate that *Atp8a2* (together with its β-subunit *Cdc50a*) plays a role in promoting neurite outgrowth^12^. Mice deficient in *Atp8a1* are characterized by impaired hippocampus-dependent learning and increased activity owing to increased PS externalization in hippocampal neurons^26^. Moreover, *ATP10C* is associated with Angelman syndrome^27^. Mutations in *ATP8B4* may play a role in Alzheimer’s disease^28^.

Similar to other P-type ATPases, P4-ATPases require heterodimeric interaction with a β-subunit to function properly. Several in vitro and in vivo studies have indicated that members of the CDC50 (also called TMEM30) protein family are required for the P4-ATPases to exit the ER and undergo transit to specific subcellular locations^29–34^. The following three members of TMEM30 family have been identified in mammals: TMEM30A, TMEM30B, and TMEM30C^35^. As the mammalian genome encoded 14 P4-ATPases and only three TMEM30 proteins, one TMEM30 protein can interact with multiple P4-ATPases. Indeed, TMEM30A is the most widely expressed β-subunit and interacts with 11 of the 14 mammalian P4-ATPases. This interaction is essential for the flippase activity and the proper localization of these P4-ATPases^29,31,32,35,36^. For example, excision of *TMEM30A* in cell lines caused a severe defect in the formation of membrane ruffles by impairing the ER exit of P4-ATPases that subsequently inhibited cell migration^29,31,37^. *TMEM30A*-deficient cells are engulfed by macrophages because of the exposure of PS in the exoplasmic leaflet, and these cells lost the ability to induce tumor formation in nude mice^13^. TMEM30A also promotes uptake of the anticancer drugs and choline phospholipids into mammalian cells^38,39^. Most recently, a number of studies have highlighted the importance of TMEM30A in vivo. In the retina, *Tmem30a* is essential for retinal photoreceptor survival^40^. In the liver, *Tmem30a* deficiency leads to intrahepatic cholestasis as a result of mislocalization of BS transporters^41^. In hematopoietic system, loss of *Tmem30a* results in rapid pancytopenia in mice and impairing leukemia cell survival^42^. However, the in vivo functions of *Tmem30a* in mammalian central nervous system remain unknown. As mutations in *ATP8A2* cause cerebellar atrophy, mental retardation, and disequilibrium syndrome in humans, this promoted us to explore the roles of *Temem30a* in the cerebellum. Indeed, considering the overlap in function and the complexity of the tissue distribution of P4-ATPases, further exploring the functions of *Tmem30a* is advisable and important.

In this study, we investigated the roles of *Temem30a* in the cerebellum by generating *Tmem30a* conditional knockout (KO) mice, in which *Tmem30a* was specifically deleted from the cerebellar PCs. *Tmem30a* KO mice displayed early-onset ataxia concomitant with cerebellar atrophy and Purkinje cell (PC) degeneration. This phenotype is most likely due to the induction of ER stress and subsequent progressive PC apoptosis in the KO cerebellum, suggested that *Tmem30a* plays a critical role in intracellular trafficking. Altogether, our studies demonstrate that *Tmem30a* is essential for the ER homeostasis and the survival of cerebellar PCs; our data also provide a direct link between *Tmem30a* functions and neurodegeneration.

## Materials and methods

### Mouse model

All animal experiments were conducted according to the guidelines of the Animal Care and Use Committee of Sichuan Provincial People’s Hospital. All experimental procedures and methods were conducted in accordance with the approved study protocols and relevant regulations. The mice were raised under cyclic lighting conditions with a 12-hour light/12-hour dark cycle.

The *Tmem30a* conditional KO mice were generated as previously described^40,41^. To generate mice with a *Tmem30a* deletion specifically in PCs of the cerebellum, *Tmem30a*^*loxP/+*^ mice were crossed with Pcp2-Cre transgenic mice (https://www.jax.org/strain/004146) to yield double-heterozygous progeny (*Tmem30a*^*loxP/+*^; Pcp2-Cre)^43^. Then, the *Tmem30a*^*loxP/+*^; Pcp2-Cre mice were crossed to *Tmem30a*^*loxP/loxP*^ animals to generate the *Tmem30a*^*loxP/loxP*^; Pcp2-Cre mice (designated *Tmem30a* KO mice). The *Tmem30a*^*loxP/loxP*^ mice were used as controls. To monitor the efficiency of the Cre-mediated deletion of the floxed exon, a tdTomato reporter gene was used (strain name: B6.Cg-Gt(ROSA)26Sortm14(CAG-tdTomato)Hze/J; also called Ai14D, http://jaxmice.jax.org/strain/007914.html)^44^. The reporter contains a loxP-flanked STOP cassette that prevents the transcription of the downstream CAG promoter-driven red fluorescent protein variant tdTomato. In the presence of Cre recombinase, the STOP cassette is removed from the Cre-expressing tissue(s) in the reporter mice. Therefore, these tissues express tdTomato.

### Genotyping by PCR

Genomic DNA samples obtained from mouse tails were genotyped using PCR to screen for the floxed *Tmem30a* alleles using the primers Tmem30a-loxP2-F (5′-ATTCCCCTTCAAGATAGCTAC-3′) and Tmem30a-loxP2-R (5′-AATGATCAACTGTAATTCCCC-3′). Pcp2-Cre was genotyped using the generic Cre primers Cre-F (5′-TGCCACGACCAAGTGACAGCAATG-3′) and Cre-R (5′-ACCAGAGACGCAAATCCATCGCTC-3′). The tdTomato mice were genotyped using the following primers provided by the JAX mouse service: oIMR9020, 5′-AAGGGAGCTGCA GTGGAGTA-3′; oIMR9021, 5′- CCGAAAATCTGTGGGAAGTC-3′; oIMR9103, 5′- GGCATTAAAGCAGCGTATCC-3′; and oIMR9105, 5′-CTGTTCCTGTACGGCATGG-3′. All amplification reactions were performed using a master mix (Invitrogen, USA).

### Histology and cell quantification

For the hematoxylin and eosin staining (H&E), deeply anesthetized animals were transcardially perfused with phosphate-buffered saline (PBS) followed by 4% paraformaldehyde. Isolated organs were fixed in 4% paraformaldehyde overnight at 4°C. Then, the fixed tissues were embedded in paraffin, cut into 5 μm sections and stained using an H&E staining protocol. The slides were imaged.

H&E-stained sections were used to measure the thickness of the molecular layer (ML) in the cerebellum and count the number of granule cells. The thickness of the ML was measured in midsagittal sections obtained from lobules IV, V, and VI (midway down the adjoining fissures) as previously described^45^. The numbers of granule cells were counted within 1000 μm^2^ areas in lobule V. All measurements were obtained from three sections from three mice of each genotype and averaged.

### Immunohistochemistry

For the immunohistochemistry, the mice were anesthetized with a combination of ketamine (16 mg/kg body weight) and xylazine (80 mg/kg body weight) and perfused transcardially with PBS, followed by 4% paraformaldehyde in 100 mM phosphate buffer (PB) (pH 7.4). The dissected heads of the animals were immersed in 4% PFA for 2 days at 4°C. Then, the brains were removed and dehydrated in 30% sucrose for 24 h. The cerebellum was embedded in optimal cutting temperature solution and sectioned at a 10 μm thickness.

For the immunohistochemistry, the sections were blocked and permeabilized with 10% normal donkey serum and 0.2% Triton X-100 in PB for 2 h and then incubated overnight at 4°C with the primary antibodies. The primary antibodies were diluted in PB containing 5% normal donkey serum and 0.1% Triton X-100 and used at the following concentrations: rabbit polyclonal ATP8A2 antibody^24,25^, TMEM30A, Cdc50-7F4 monoclonal antibody (supernatant 1:3, a gift from Dr. Robert Molday, University of British Columbia, Canada); Calbindin-D28K (Sigma, St. Louis, MO, USA); cleaved caspase-3, C/EBP homologous protein (CHOP) and BiP (Cell Signaling Technology, CA, USA); Glial fibrillary acidic protein (GFAP) (Santa Cruz Biotechnologies, CA, USA); PKC-γ (Abcam, Cambridge, MA, USA). The sections were incubated with the primary antibodies overnight. Then, the sections were washed three times with PBS, incubated for 1 h with Alexa-Fluor-488- or Alexa-Fluor-594-labeled goat anti-mouse or rabbit Ig secondary antibodies (diluted 1:500) and counterstained with 4′,6-diamidino-2-phenylindole. terminal deoxynucleotidyl transferase dUTP nick end labeling (TUNEL) was performed using an In Situ Cell Death Detection Kit (Roche, Redwood City, CA, USA). The images were captured under a Zeiss LSM 800 confocal scanning microscope.

Quantification of the PCs was performed using Calbindin immunostaining to identify the PCs. All PCs in fields from midsagittal sections were counted, and the results obtained from three sections were averaged.

### Immunocytochemistry

COS7 cells were cultured in Dulbecco’s modified Eagle’s medium/GlutaMAX (Invitrogen, CA, USA) supplemented with 10% fetal bovine serum (Invitrogen) and 100 units/ml penicillin/streptomycin (Invitrogen) under 5% CO_2_ at 37 °C. The cells were transiently transfected with HA-tagged TMEM30A or Flag-tagged ATP8A2. After 2 days, the transfected cells were fixed with 4% paraformaldehyde, and the TMEM30A proteins were visualized using rat anti-HA antibody (Roche, Redwood City, CA, USA). The ER marker proteins were labeled with a rabbit anti-Calnexin antibody (Cell Signaling Technology, CA, USA), the Golgi markers were labeled with a rabbit anti-GM130 antibody (BD Biosciences, Mississauga, ON). The early endosomes were labeled with an antibody against the specific marker Rab11a (BD Biosciences, Heidelberg, Germany). The secondary antibodies used included goat anti-rabbit Alexa 488, goat anti-rabbit Alexa 594, goat anti-mouse Alexa 488, goat anti-mouse Alexa 594, donkey anti-rat Alexa 488 and donkey anti-rat Alexa 633 (Invitrogen). The images were captured under a Zeiss LSM 800 confocal scanning microscope.

### Immunoblotting

The tissues were lysed in RIPA lysis buffer (50 mM Tris-HCl, 150 mM NaCl, 1%Triton X-100, 0.5% sodium deoxycholate, 0.1% SDS, pH 7.4) supplemented with Complete Protease Inhibitor Cocktail (Roche), and the protein concentration of the lysates was determined using a DC Protein Assay (Bio-Rad). Equal amounts of protein were separated on SDS polyacrylamide gels and transferred to PVDF membranes (GE Healthcare, Chicago, IL, USA). The blots were blocked with 8% non-fat dry milk in TBST for 2 h at room temperature. Then, the membranes were incubated with the primary antibodies in blocking solution overnight at 4°C. The following primary antibodies were used for the Western blotting: antibodies against CHOP and BiP, cleaved caspase-3, cleaved caspase-12 and GAPDH (Cell Signaling Technology, Danvers, MA, USA) and an antibody against β-actin (Proteintech Group, Chicago, IL, USA). The primary antibodies were detected with anti-mouse or anti-rabbit HRP-conjugated secondary antibodies (1:5000; Bio-Rad, Hercules, CA, USA), and the signal was developed using Supersignal West Pico Chemiluminescent Substrate (Pierce). The relative intensity of the immunoreactive bands was quantified using the gel analysis tool provided in the ImageJ software. The normalization of the proteins of interest was performed relative to GAPDH or β-actin.

### Behavioral analysis

For the hindlimb clasping assessment, the mice were lifted by their tails and held over a cage for up to 2 min to assess abnormal hindlimb clasping. Clasping was scored when the animal crossed its hindlimbs for more than 3 s. The rotarod test was performed by placing 2-month-old mice on a rod that accelerated from 5 rpm to 30 rpm over a period of 2 min. The latency to fall off the rod was measured. The rotarod apparatus (Unibiolab, Shanghai, China) was used to evaluate the motor performance of animals. Only male mice were used in the behavioral experiments.

### Transmission electron microscopy (TEM)

Anesthetized mice were fixed by transcardial perfusion with PBS, followed by perfusion with 2.5% glutaraldehyde in cacodylate buffer. Brains were dissected and post fixed in 2.5% glutaraldehyde in cacodylate buffer, pH 7.2, overnight at 4°C. The cerebella were dissected from the heads, immersion-fixed for 8 h, and sectioned sagittally at 100-mm intervals using a vibratome. The cerebellar sections were incubated in 1% osmium tetroxide for 1 h, washed in 0.1 M phosphate buffer, dehydrated via an ascending series of ethanol and propylene oxide, and embedded in Epon (25 g Epon 812, 13 g dodecenyl succinic anhydride, 12 g nadic methyl anhydride, and 1 ml 2,4,6-Tris (dimethylaminomethyl) phenol (DMP-30), Electron Microscopy Sciences). Ultrathin sections (70 nm) were cut and stained with uranyl acetate and lead citrate. The sections were imaged under a Philips CM120 scanning transmission electron microscope.

### Gene expression analysis by reverse transcription PCR (RT-PCR)

Total RNA was extracted from male mice cerebellum tissue using TRIzol reagent (Invitrogen, Waltham, MA, USA) according to the manufacturer’s instructions. RNA samples were treated with RNase-free DNaseI (Ambion, Austin, TX, USA) to remove genomic DNA. The complementary DNA was synthesized by MessageSensor RT kit (Ambion, TX, USA) with a total of 3 µg of RNA and random primers. Primer sequences were listed in Table S1. PCR was performed with Taq polymerase (New England Biolabs, MA, USA), and the PCR products were resolved on 3% agarose gels.

### Statistical analysis

The statistical significance was performed using Student’s *t* test or one-way analysis of variance. The differences were considered statistically significant at *P* values < 0.05. The quantitative data are presented as the mean ± SEM as indicated in the figure legends. The statistical analyses were performed using GraphPad Prism 6 software. The results of the statistical tests are described in the individual figure legends.

## Results

### Expression of *Tmem30a* in PCs

*Tmem30a* is expressed in the retina, brain, cerebellum, liver, heart, kidney, spine, and testis^32,34,40^. To elucidate the functions of *Tmem30a* in cerebellar PCs, we first assessed the expression pattern of *Tmem30a* in the mouse cerebellum. Sections of cerebellum were immunostained with a specific antibody against TMEM30A, and PKC-γ served as a marker of the PCs^46^. The specificity of the mouse monoclonal antibody against TMEM30A has been previously verified^40^. *Tmem30a* is widely expressed in the cerebellum, in PKC-γ positive PCs as early as postnatal day 16 (P16) and in adulthood (Fig. S1). TMEM30A signals were strong in the ML and PC layer (PCL) of the cerebellar cortex, where PC soma and dendrites are localized, and the signals were relatively weak in the granule cell layer (GCL) (Fig. S1). Thus, *Tmem30a* may play pivotal roles in the development and survival of PCs.

### Loss of *Tmem30a* in PCs causes early-onset ataxia and cerebellar atrophy

**Fig. 1:**
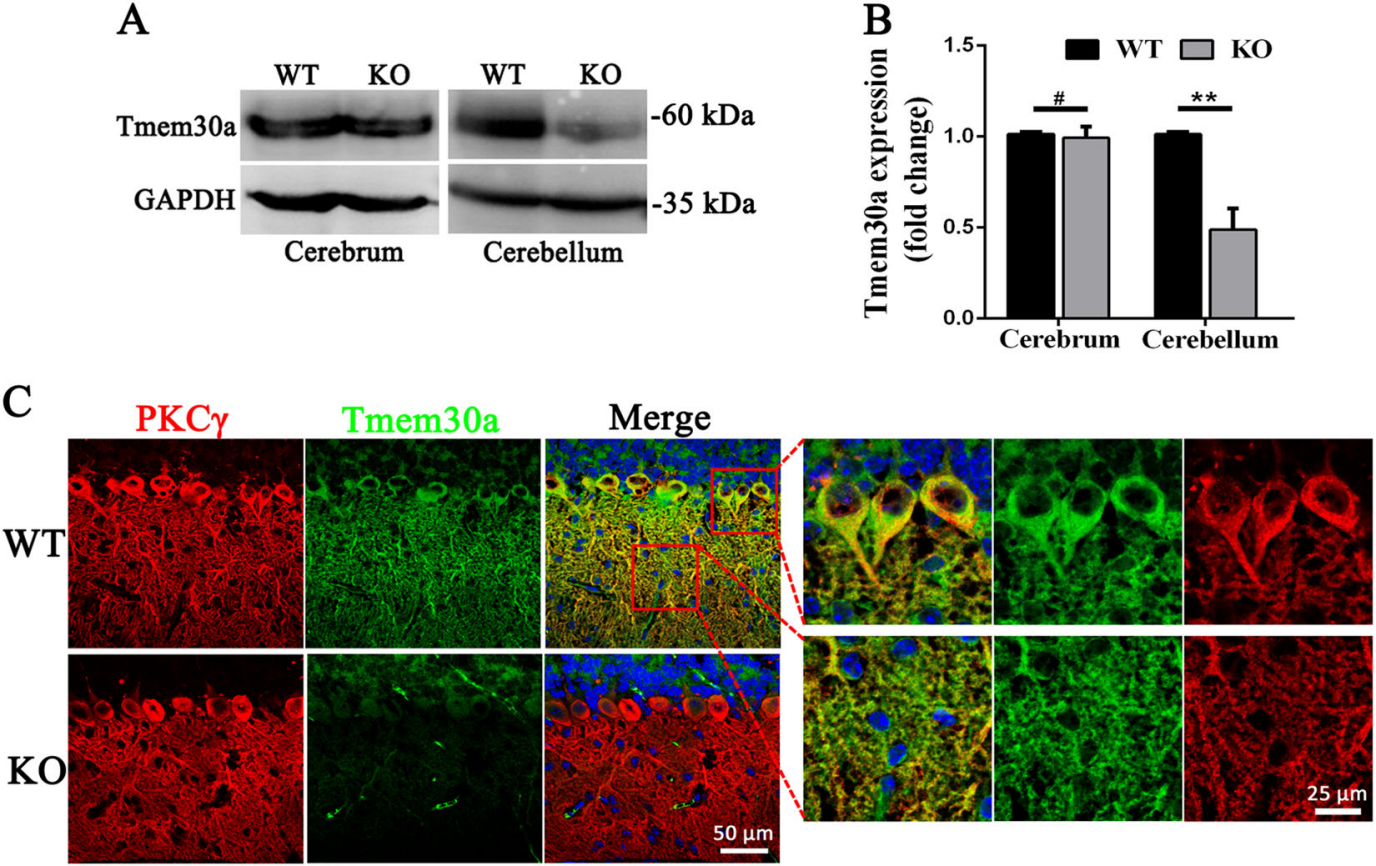
*Tmem30a* was removed from cerebellar PCs in KO mice. **a**–**b** Reduced expression levels of Tmem30a protein in the cerebellum of KO mice (1 month old), as shown by western blot analysis of total lysates of the cerebrum and cerebellum from WT and KO mice using anti-TMEM30A antibody. Cerebrum samples were used as control. *N* = 3. **, *p* < 0.01. The data represented means ± SEM. **c** Immunofluorescence labeling of cerebellum cryosections from WT and KO littermates at P23 using anti-TMEM30A antibody (red), PKC-γ antibody (green), and DAPI (blue). PKC-γ was used to label PCs. Strong signals of Tmem30a in the molecular and Purkinje cell layer were observed in WT but reduced level of Tmem30a in the KO cerebellum

To investigate the role of *Tmem30a* in cerebellar PCs, we generated *Tmem30a* PC-specific KO mice by crossing L7 promoter (Pcp2-Cre) transgenic mice with *Tmem30a*^*loxP/+*^ mice in which exon 3 was flanked by loxP sites as previously described^45,46^ (Fig. S2A). PCR genotyping was performed to distinguish the homozygous *Tmem30a* KO mice from their wild-type (WT) and heterozygous (Het) littermates (Fig. S2B). In addition, to verify the specific expression of Pcp2-Cre, we crossed ROSA26-tdTomato reporter mice with *Tmem30a*^*loxP/loxP*^; Pcp2-Cre mice to generate *Tmem30a*^*loxP/loxP*^; Pcp2-Cre; Rosa-tdTomato mice. In the presence of the Cre enzyme, the stop codon before the tdTomato expression cassette is removed, resulting in the expression of tdTomato in the Cre-positive cells (Fig. S2C). We labeled the tdTomato-expressing cells using antibodies against the specific marker Calbindin. The tdTomato-expressing cells in the cerebellum were distinctly immunoreactive for Calbindin, indicating that Pcp2-Cre expression was specific in PCs (Fig. S2C). To verify the efficiency of *Tmem30a* excision in PCs, we prepared brain and cerebellar lysates from adult *Tmem30a* KO mice and subjected these brain lysates to immunoblotting analysis (Fig. 1a). The expression level of *Tmem30a* in the *Tmem30a* KO cerebellum was reduced to ~ 46% of that in the WT littermates, whereas *Tmem30a* expression in the KO cerebral cortex did not change (Fig. 1b). We confirmed this by immunofluorescence staining of TMEM30A and Calbindin. TMEM30A signals were strong in the WT PCs as mentioned above but were almost absent in the KO PCs (Fig. 1c).

**Fig. 2:**
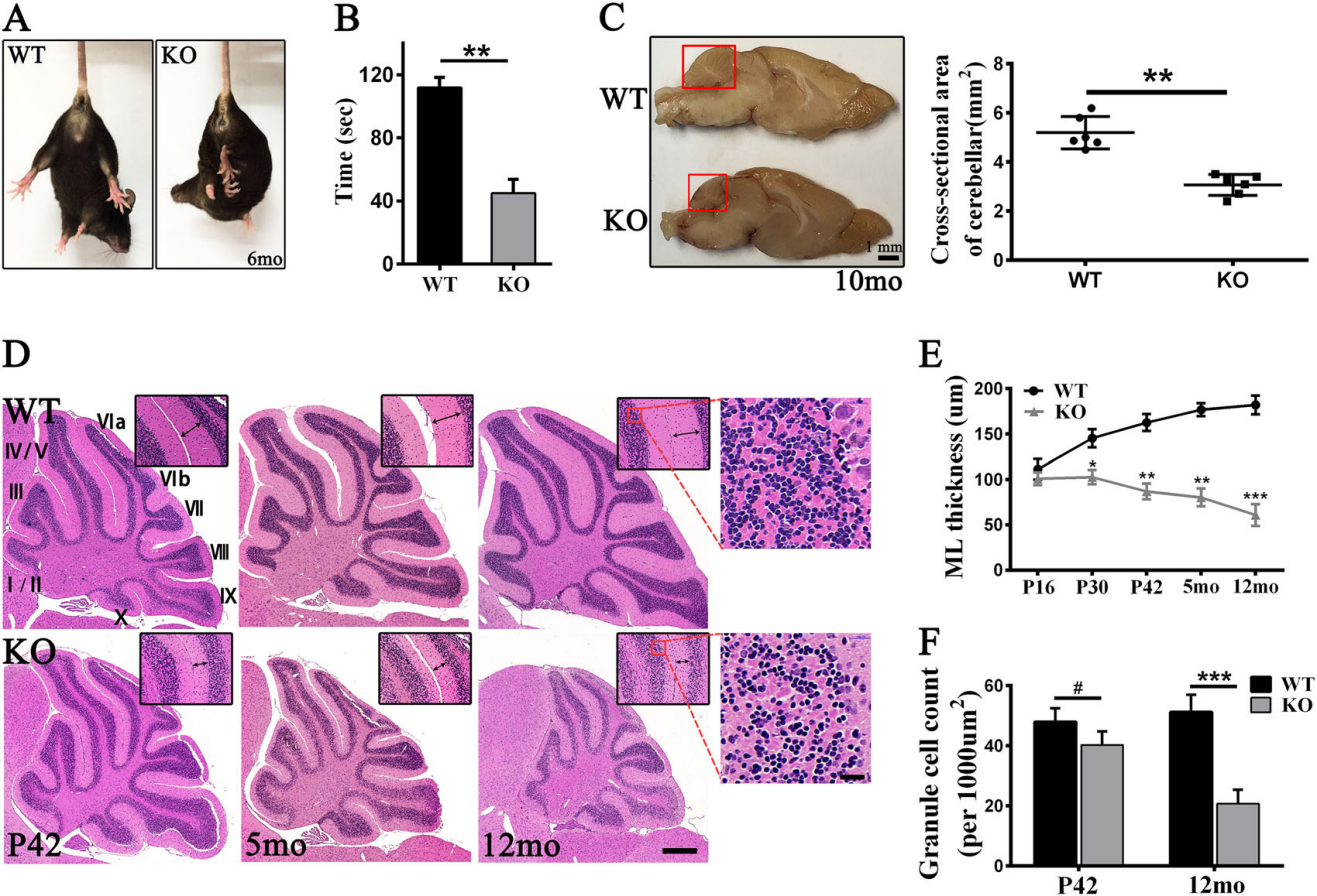
Ataxia phenotypes and cerebellar atrophy in *Tmem30a* KO mice. **a** Hindlimb clasping phenotype of *Tmem30a* KO but not WT mice at 6 month of age during tail suspension. **b** Differences in accelerating rotarod performance observed between WT (*n* = 9) and KO mice (*n* = 8) were statistically significant (**, *p* < 0.01). **c** Photograph of midsagittal sections of brains from 10 month KO and control mice. Quantification of the cross-sectional area of cerebellum (red boxed area). *n* = 3 mice for each genotype (**, *p* < 0.01). **d** Hematoxylin and eosin stained sagittal sections of cerebella from WT and KO mice in P42, 5 month and 12 month. Higher-magnification images from lobule V/VI are shown in the upper right corner of each picture. ML thickness of each genotype in different ages was indicated by double-sided arrows. The right images exhibited the higher-magnification images of boxed areas in the left panels. Lobules were indicated by Roman numerals. ML, Molecular layer. **e** Quantitative assessment of the thickness of the molecular layer of WT and KO mice in different ages. Sample size *n* = 5; *, *p* < 0.05; **, *p* < 0.01; ***, *p* < 0.001. **f** Quantitative analysis of the number of GC in WT and KO mice (*n* = 5–7; ***, *p* < 0.001). The data represent means ± SEM

*Tmem30a* KO mice were born at Mendelian ratios and could not be distinguished from their littermate controls at birth. At P20, the KO mice exhibited mild ataxia and tremor reminiscent of *Atp8a2* mutant mice^25,47^, whereas the heterozygous mice were indistinguishable from their WT littermates. By 3 months, the KO mice were extremely ataxic and unable to walk smoothly. The KO mice were subjected to behavioral tests, including tail suspension test and rotarod test. Once suspended by their tails, the KO mice exhibited clasped hindlimbs, which is a typical phenotype of neurologically defective mice (Fig. 2a). Rotarod test revealed that motor coordination was significantly impaired in the KO mice (Fig. 2b).

The cerebellum is the main control center of motor coordination. The cerebella of the KO mice displayed severe atrophy from 5 months onward (Fig. 2d). Midsagittal sections obtained from KO mice of 10 months old showed that the cerebellar area was reduced by ~ 43% in these animals but did not reveal any obvious alterations in other brain regions (Fig. 2c). These results were confirmed by H&E staining of the cerebellar cortices of the KO mice from P30 to 12 months of age (Fig. 2d). Based on the measurement of the thickness of the ML of the cerebella of mice at different ages, the onset of cerebellar atrophy occurred at approximately P30 (Fig. 2e). The thickness of the ML in KO cerebellum, in which the dendrites of PCs are located, was reduced by ~ 72% at 12 months of age (Fig. 2e). Importantly, a prominent loss of PCs was observed at P30, which is the time at which the compression of the ML began to occur in the KO cerebellum, indicating that the compression of the ML was owing to PC loss. Moreover, loss of cells from the internal granular layer might also contribute to the macroscopic cerebellar atrophy observed at subsequent times (Fig. 2d–f). In contrast, the cerebella from the *Tmem30a* heterozygous animals appeared structurally normal compared with those from their age-matched control littermates (Fig. S3A, B).

### PC degeneration and astrogliosis in *Tmem30a* KO mice

**Fig. 3:**
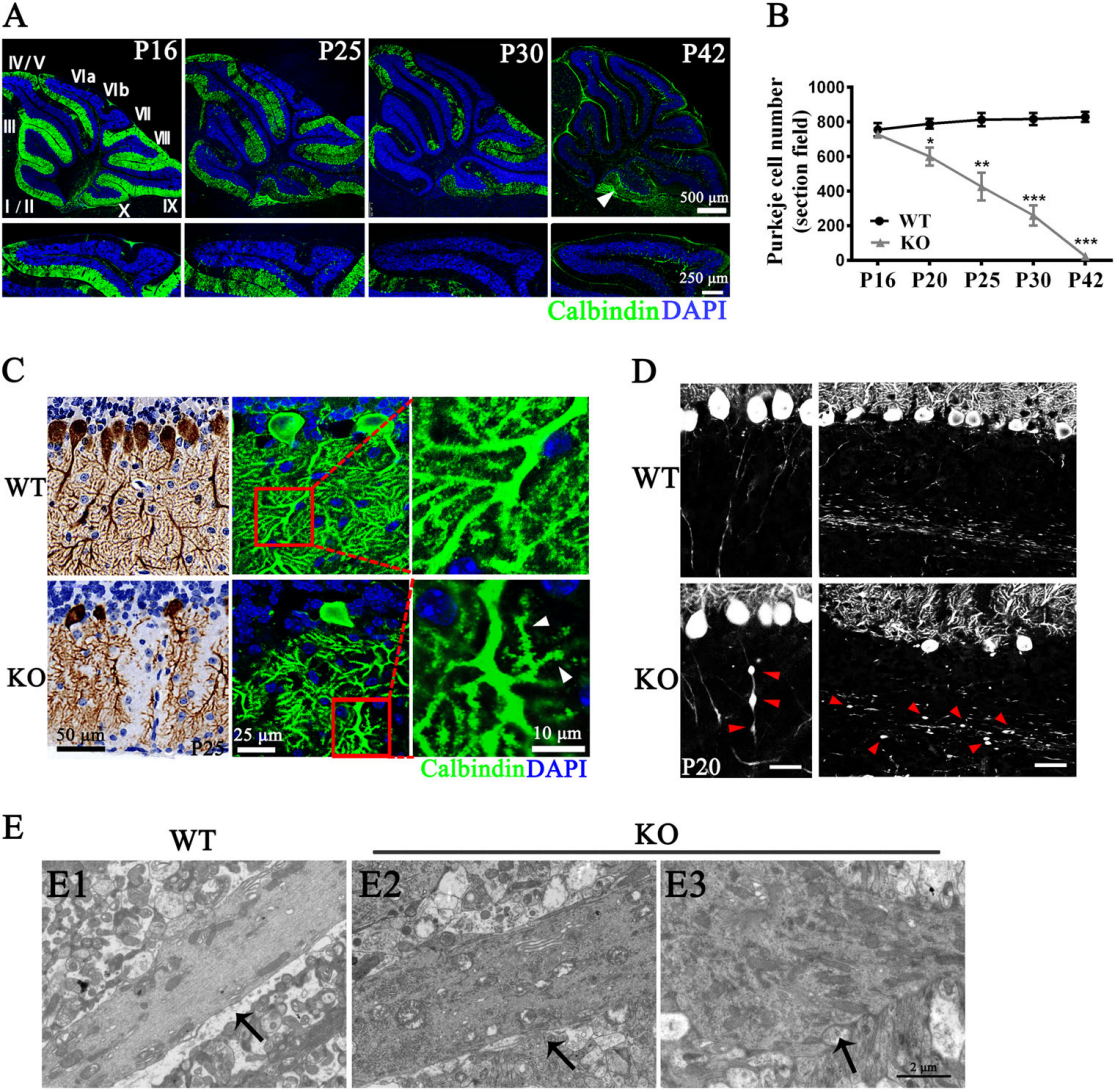
Degeneration of Purkinje cells in KO mice. **a** Immunofluorescence labeling of cerebellar cryosections of WT and KO littermates at P16, P25, P30, and P42 using Calbindin antibody (green) and DAPI (blue). Higher-magnification images from lobule VI/VII were shown in the low panel. Arrows pointed to lobule X with no obvious Purkinje cell loss. Lobules are indicated by Roman numerals. **b** Quantification of the Calbindin labeled Purkinje cells in different ages (P16, P20, P25, P30, and P42). The number of PC declined from P20 in *Tmem30a* KO. *n* = 5; *, *p* < 0.05; **, *p* < 0.01; ***, *p* < 0.001.The data represent means ± SEM. **c** Calbindin immunostaining (left: DAB staining) revealed loss of dendrites in *Tmem30a* KO Purkinje cells in lobule V with loss of PC dendrites (left) and deformed, degenerating spiny branchlets (right, arrows) compared with WT control. Boxed areas at the right were shown at higher-magnification as middle insets. **d** Calbindin immunostaining further revealed axonal spheroids (torpedoes) in the initial axonal segment (left, arrows) and white matter (right, arrows) of KO cerebellum, indicating cell degeneration. **e** Representative transmission electron microscope (TEM) images of axons in the P16 WT (E1) and KO (E2, E3) white matter in the cerebellar cortex. Arrow in E1 indicates normal WT axon. Arrows in E2 and E3 indicate representative degenerative axons

To further confirm that PC degeneration occurs in the KO cerebellum, sections of cerebella obtained from mice at different ages were immunostained for Calbindin to mark PCs. We examined the cerebellum at P7 to determine whether any of the structural changes could be observed at early stages of development. No significant differences were observed between the WT control and KO mice at P7 (Fig. S4A). In contrast, at P9, characteristics of dystrophic neurons, including shrunken soma and developmental retardation of dendrites, were observed, but no obvious PC loss was evident at this stage (Fig. S4B). PC loss was first detected in the KO cerebellum at P16, and PC degeneration became more pronounced over time (Fig. 3a). At P25, ~ 50% of the PCs had been lost, which is consistent with the reduced thickness of the ML and the early onset of ataxia in the KO mice (Fig. 3b). Interestingly, except for the PCs in lobule X, no PCs survived after P42 (Fig. 3a, arrows). Considering that *Tmem30a* was also deleted in lobule X, metabolic differences might exist between lobule X and other parts of the cerebellum^47^. Calbindin immunolabeling also revealed the details of the dendritic and axonal degeneration in the KO mice during the early period of PC degeneration. The DAB staining of paraffin sections of the Tmem30a KO cerebella showed extensive degeneration of the dendrites prior to the cell body loss at P25, including a decrease in soma size and a marked reduction in the region occupied by dendrites in the KO cerebellum (Fig. 3c). However, these features of PC degeneration were absent in the *Tmem30a* heterozygous mice (Fig. S3A, C).

An examination of the PC dendrites at a high magnification revealed a substantial decrease in dendritic spine density and significant morphological changes in both the apical and basal dendrites of the KO PCs (Fig. 3c, arrows). These changes resulted in the loss of synaptic contacts between the PCs and target neurons. Calbindin immunolabeling also identified axonal swellings in the internal GCL and within the core of the cerebellar white matter in the KO mice (Fig. 3d, arrows). Such swellings are considered a hallmark of axonal dystrophy^48^. In contrast, the axons in the cerebella from the WT mice were slender and displayed a uniform caliber (Fig. 3d, left). The PC axons in the KO mice also degenerated as shown by the reduced number of axons present in the white matter in the KO mice compared with that in the control cerebella (Fig. 3d, right). To further confirm the degeneration of the PC axons, the cerebellar GCL tracts in the KO and WT mice were examined by TEM. The electron micrographs revealed axonal pathologies, including the accumulation of organelles in swollen nerve fibers (Fig. 3E3), the presence of vacant areas, and autophagosome-like bodies owing to degeneration of nerve fibers (Fig. 3E2). These changes were widespread in the white matter of the KO cerebella but completely absent from the same regions of the WT cerebella (Fig. 3E1), confirming the immunofluorescence results mentioned above. The degeneration of dendrites and axons in the KO PCs subsequently cause a dysfunction in the PCs.

**Fig. 4:**
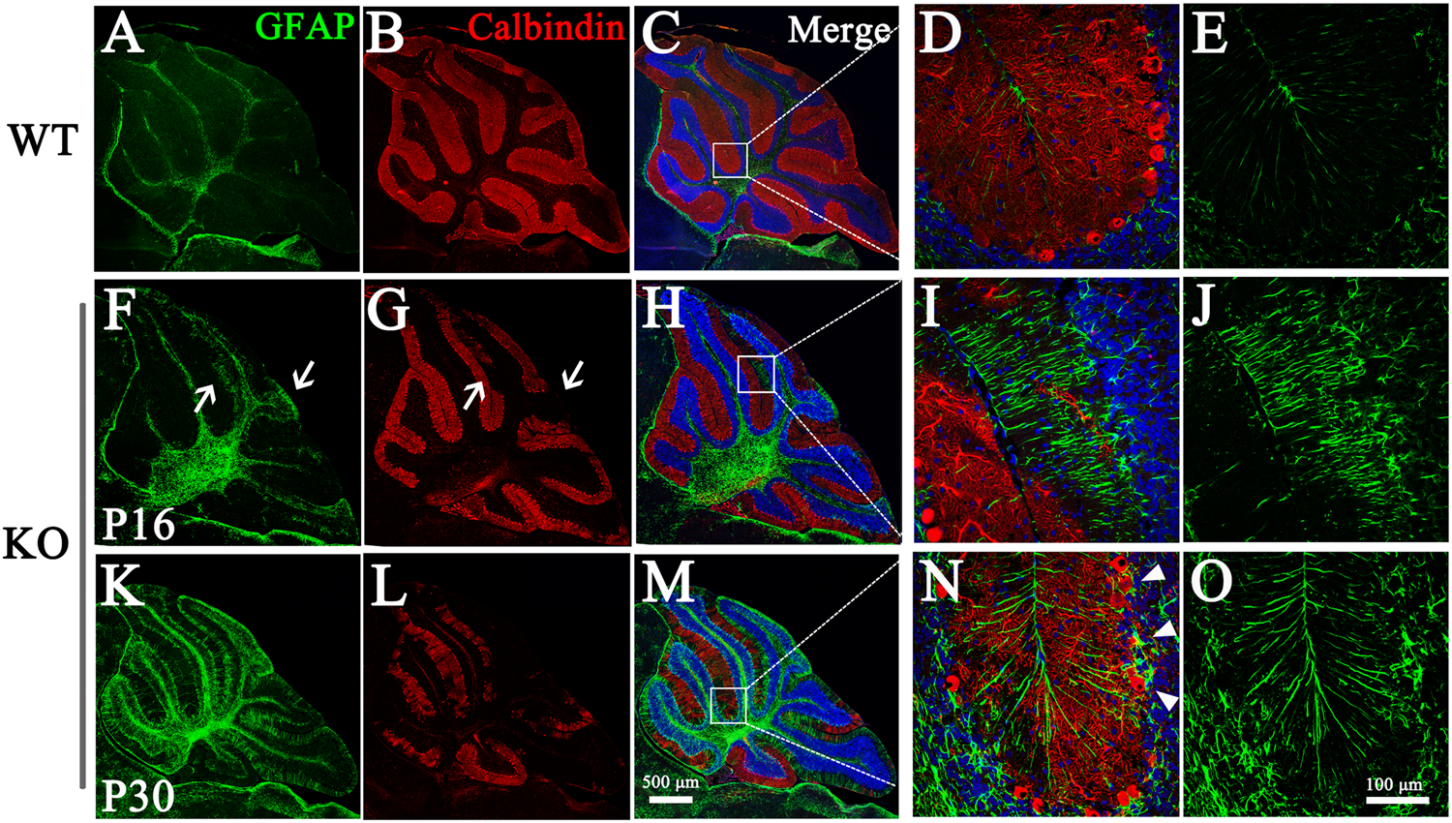
Astrogliosis in the degenerating *Tmem30a* KO cerebellum. Sections from cerebellum of control **a**–**e** and KO **f**–**o** mice were co-staining for the PC marker Calbindin (red) and the astrocyte marker GFAP (green) at P16 **f**–**j** and P30 **k–o**, two time points when PCs exhibited initial and severe degeneration, respectively. The right images **d**, **e**, **i**, **j**, **n**, **o** exhibited the higher-magnification images of boxed areas in the left panels **c**, **h**, **m**. A growing number of astrocytes were activated concomitant with the increasing PC degeneration from P16 to P30 **a**, **f**, **k**. Arrows: astrogliosis appeared at the area where prominent Purkinje cell had lost. WT cerebellum showed parallel Bergmann glia processes with weak GFAP **a**–**e**, whereas intensely stained, thickened, and unorganized processes in KO cerebellums **i**, **j**, **n**, **o**. Arrowheads: activated Bergmann glia with increased GFAP expression and inceptive signs of disorganization in the area where no loss of PCs occurred yet. Merged images show DAPI co-staining (blue)

Bergmann glia, which constitute a specific type of radial astrocyte in the cerebellum, display a unique interaction with PCs. The cell bodies of Bergmann glia are located in the PCL, and Bergmann glial fibers extend into the ML, where they enwrap synapses on PC dendrites. This close interaction is important for PC survival as well as damage repair^49–52^. Under pathological conditions, astrocytes are rapidly activated and able to exert neuroprotective and toxic effects. Astrocytes were also assessed in this study. To this end, cerebellar sections obtained from mice at different ages were immunostained with GFAP as a marker of astrocytes. In the WT cerebellum, parallel fibers with low GFAP immunoreactivity crossed the ML (Fig. 4a–e). By contrast, in the degenerated areas in the KO cerebellum, the Bergmann glial processes in the ML were thicker, appeared disorganized and were more intensely immunoreactive for GFAP (Fig. 4f–o), indicating astrogliosis-like activation of the Bergmann glia. Using GFAP staining, astrogliosis was detectable at P16 and became much more severe at P30 (Fig. 4f–k), which is consistent with the degeneration pattern of PCs. Notably, astrogliosis appeared specifically in areas in which prominent PC loss had occurred (Fig. 4f–j, arrows). Interestingly, in areas without extensive PC degeneration, individual Bergmann glia with increased expression of GFAP and disorganized processes were occasionally observed (Fig. 4n, arrows), suggested that the activation of Bergmann glia preceded the PC loss and might accelerate the degeneration of PCs.

### TMEM30A is involved in ER-Golgi and/or intra-Golgi trafficking in COS7 cells

Previous studies investigating the overexpression of *Tmem30a* in CHO cells and U2OS cells suggested that this protein is predominantly localized in the ER^31,39,53^. Consistent with these earlier studies, ER localization was observed after the transfection of COS7 cells with a plasmid encoding HA-tagged human *TMEM30A*. The immunostaining of the transfected cells with antibodies against the HA tag yielded a reticular distribution, suggesting ER localization. This was confirmed by co-immunostaining with antibodies against Calnexin, an ER marker (Fig. S5A–D). A strong signal was also observed surrounding the nucleus as expected based on the ER membrane localization of TMEM30A. The GM130 staining of the transfected cells also revealed that TMEM30A was partially localized in the Golgi complex (Fig. S5E–H). Confocal imaging of the transfected cells showed that a relatively high level of TMEM30A immunofluorescence colocalized with the recycling endosome marker Rab11a (Fig. S5I–L), which is a small GTPase associated with intracellular membrane trafficking. Collectively, TMEM30A likely has a fundamental role in vesicle-mediated protein transport in intracellular membrane systems.

### *Tmem30a* loss in PCs causes ER stress and apoptotic cell death

**Fig. 5:**
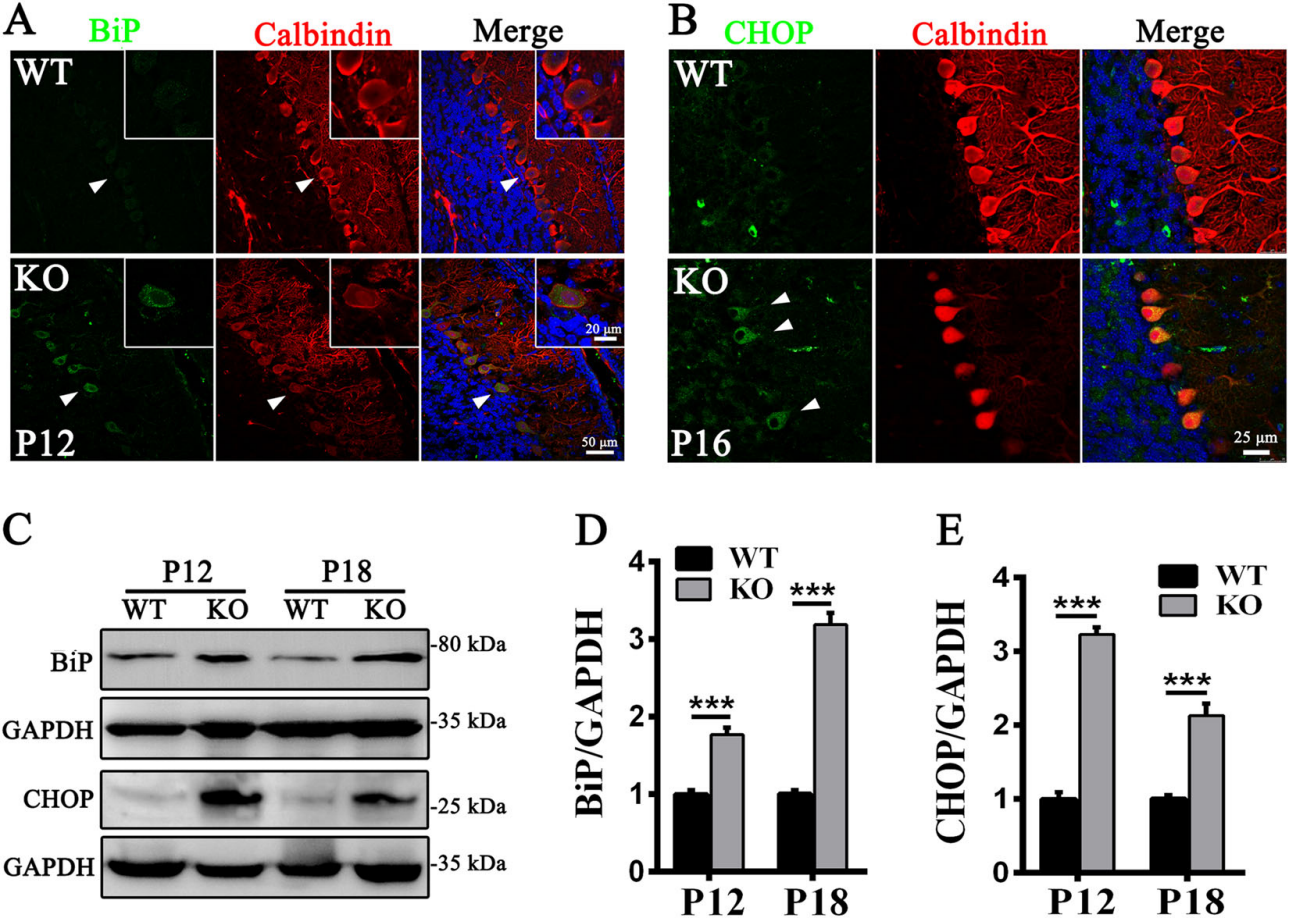
Loss of TMEM30A function induces ER stress in Purkinje cells. **a** ER stress was evaluated by co-immunostaining with ER stress marker BiP and PC marker Calbindin in cerebellar sections from P12 WT and KO mice. BiP (green) positive staining (arrowheads) was observed in the PCs of *Tmem30a* KO mice cerebellum but absent from the WT cerebellum. Higher-magnification images of representative BiP positive staining PC (arrowheads) shown in the top right of each picture. **b** Cerebellar sections from P16 WT and KO mice were immunostained with antibodies to CHOP (green) and Calbindin (red). Arrowheads indicate CHOP positive staining. **c**–**e** Western blots **c** and quantitation **d**–**e** of cerebellar protein extracts from P12 and P18 wild-type and KO mice, respectively, probed with an antibody against ER stress markers BiP and CHOP, GAPDH antibody as loading control. Sample size *n* = 6, ***, *p* < 0.001. The data represent means ± SEM

To investigate the mechanisms leading to progressive PC degeneration, we performed a detailed analysis of the ER stress response owing to the vital function of *Tmem30a* in intracellular trafficking. The expression of representative ER stress markers was examined in WT and KO mice at P12 prior to the appearance of major phenotypic defects. BiP (GRP78), a major HSP70 chaperone of the ER lumen and a central regulator of ER homeostasis. Co-immunofluorescence with antibodies against BiP and Calbindin demonstrated that BiP was strikingly upregulated around the nuclei in the KO PCs (Fig. 5a). Moreover, the major mediator of ER stress for apoptosis, CHOP was also upregulated in a higher number of PCs in the KO cerebella (Fig. 5b). These results were confirmed by immunoblotting. The expression levels of both BiP and CHOP were considerably elevated during the early stage of degeneration (Fig. 5c–e), implying the occurrence of ER stress response in the KO PCs. Notably, the predominant death of Purkinje neurons was absent during this stage, indicated that ER stress occurred prior to neurodegeneration in the KO cerebellum.

**Fig. 6:**
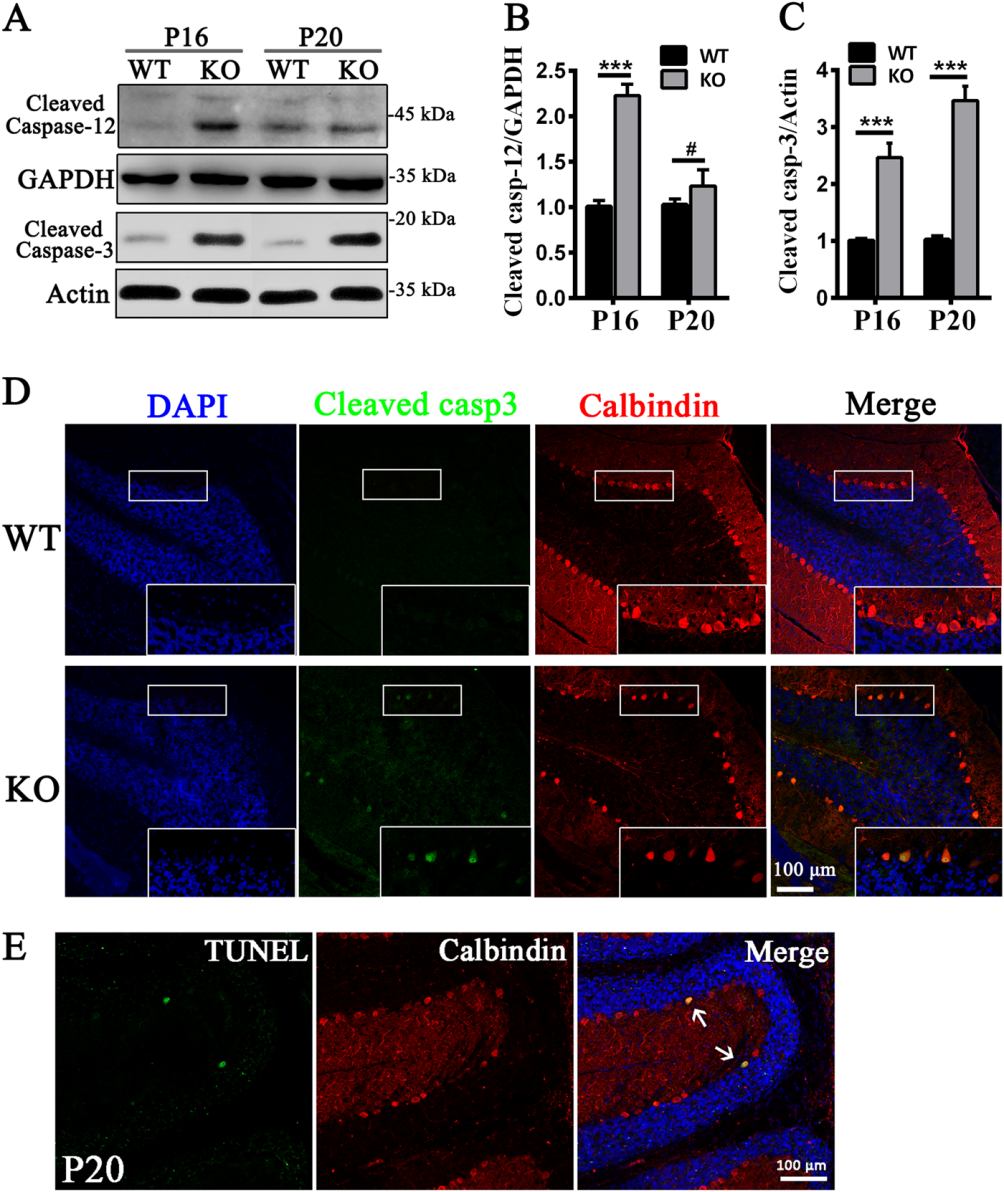
Activation of apoptosis in *Tmem30a* KO mice. **a**–**c** Western blots **a** and quantitation **b**, **c** manifested the elevated expression of cleaved caspase-12 and cleaved caspase-3 in cerebellum of WT and KO mice at P16 and P20, respectively, and GAPDH antibody as loading control. (*n* = 6,***, *p* < 0.001).The data represent means ± SEM. **d** Immunofluorescence labeling of cerebellum cryosections from P20 control and mutant littermates with cleaved caspase-3 (green) and Calbindin (red). Cleaved caspase-3 staining positive cells increased dramatically in mutant mice. **e** Detection of apoptotic Purkinje cells in KO cerebellum at P20 using the TUNEL assay. Antibody to Calbindin was used to mark Purkinje cells. Arrows represent TUNEL-positive cells

Owing to the activation of the ER stress pathway in the KO cerebellum, we examined the apoptotic signaling pathway in the KO cerebellum. Cleaved caspase-12, which is a typical ER stress-induced apoptosis marker, was upregulated at P16 in the KO cerebellum (Fig. 6a, b), suggested that the prolonged ER stress triggered the caspase cascade before cell apoptosis. Moreover, both immunofluorescence and immunoblotting results demonstrated that the expression level of cleaved caspase-3, which is an important marker of cell apoptosis, was elevated from P16 to P20 (Fig. 6a–d). This is consistent with the rapid cell loss that occurred after P16 (Fig. 3a, b). In addition, the number of TUNEL-positive cells in the KO cerebella at P20 was higher (Fig. 6e, arrows) than that in the control cerebella. Collectively, loss of *Tmem30a* in PCs induced the activation of the ER stress pathway and subsequent cell apoptosis, explaining the rapid loss of PCs in the KO cerebellum.

### TMEM30A acts with other flippases but not ATP8A2 in PCs

**Fig. 7:**
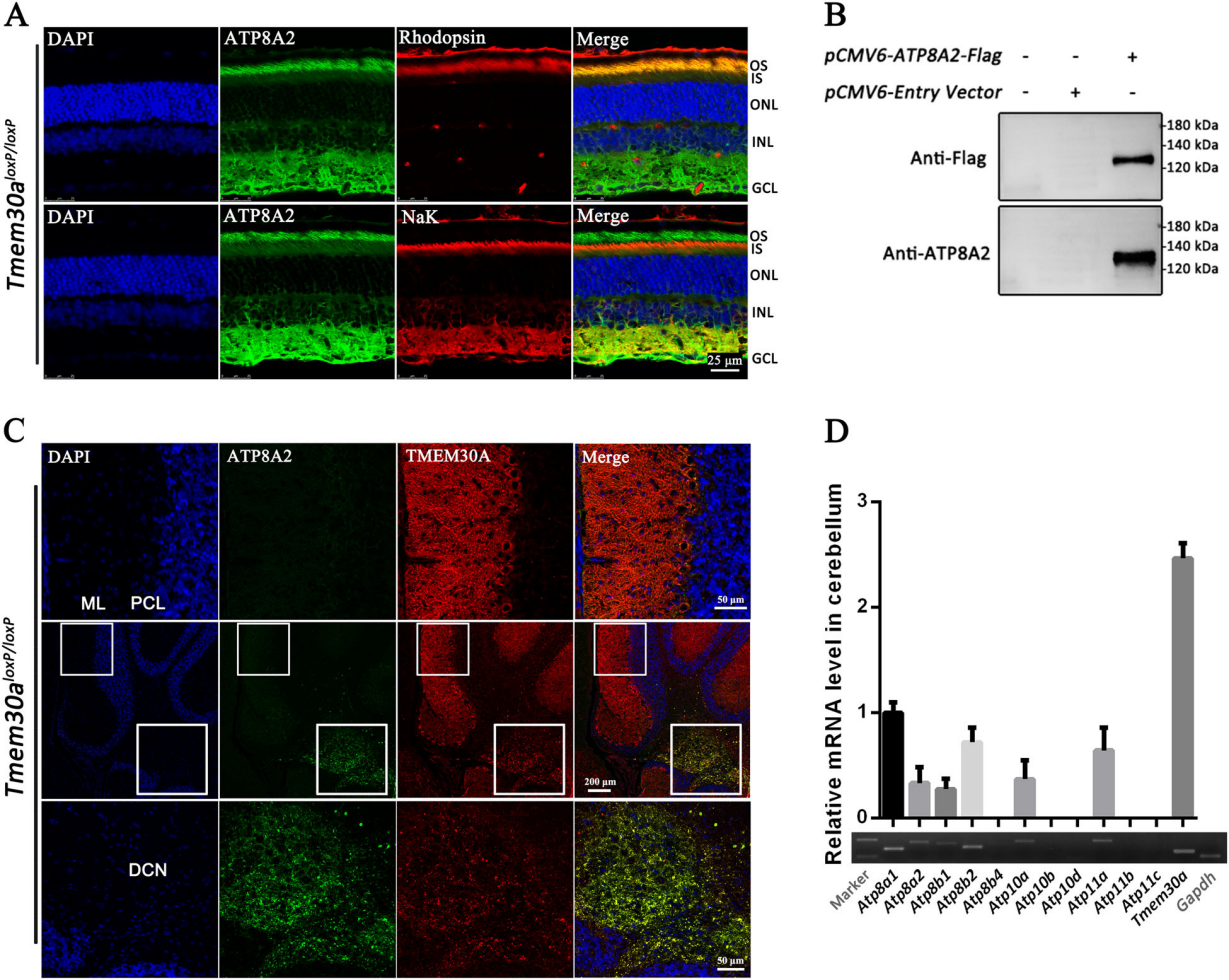
TMEM30A acts with other flippases but not ATP8A2 in PCs. **a** ATP8A2 antibody specifically detects ATP8A2 expression in the outer segment of the retina. The antibody was evaluated by co-immunostaining with retinal outer segment (OS) marker Rhodopsin and inner segment (IS) marker NaK, respectively, in the retina sections from *Tmem30a*^*loxP/loxP*^ mice. ONL, outer nuclear layer; INL, inner nuclear layer; GCL, ganglion cell layer. **b** ATP8A2 antibody detects a band of 130 Kd (ATP8A2 protein) in western blotting analysis. In total, 293 T cells were transiently transfected with an expression vector for C-terminally Flag-tagged *ATP8A2* or/and a empty pCMV6 expression vector. The empty pCMV6 expression vector was used as a negative control. Cell lysates were immunoblotted using anti-Flag and anti-ATP8A2 antibodies. **c** ATP8A2 is not expressed in PCs but in deep cerebellar nucleus (DCN). Immunofluorescence labeling of cerebellum cryosections from *Tmem30a*^*loxP/loxP*^ mice was performed using TMEM30A antibody (red), ATP8A2 antibody (green), and DAPI (blue). Higher-magnification images were shown in the upper/low panel. Strong signals of TMEM30A in ML and PCL were observed (upper panel), whereas ATP8A2 were only observed in the deep cerebellar nuclei (DCN) (lower panel). **d** Multiple PS flippases are expressed in the cerebellum. Total RNA of mouse cerebellum was extracted. Reverse transcription PCR analysis was performed to evaluate the expression levels of PS flippases in mouse cerebellum (*n* = 4). *Gapdh* was used as a control. The data represent means ± SEM

Previous studies have described the interaction between ATP8A2 and TMEM30A^29,31,34,35^. Given the phenotypic similarity of *Tmem30a* KO mice and *Atp8a2* mutant mice^24^, we assessed the expression of ATP8A2 in the cerebellum. No positive expression was observed using the anti-ATP8A2 antibody described previous^24,25^. This antibody specifically detected ATP8A2 in the outer segment of the retina and western blotting analysis (Fig. 7a, b). Immunofluorescence labeling of cerebellum cryosections from *Tmem30a*^*loxP/loxP*^ mice using ATP8A2 antibody revealed no ATP8A2 expression in PCs (Fig. 7c, upper panel). ATP8A2 is expressed in deep cerebellum nuclei (DCN) (Fig. 7c, lower panel). This result is in consistence with our previous study using *Atp8a2* mutant mice^24^. In *Atp8a2* mutant mice, central chromatolysis, which indicated axonal injury, was observed in the deep cerebellum nuclei^24^. Neurons in the DCN received input from PCs. Loss of *Atp8a2* in DCN led to degenerated neurons, which caused ataxia-like phenotypes, similar to that of *Tmem30a* KO.

We assessed expression of PS flippases in the mouse cerebellum by RT-PCR (Fig. 7d). *Tmem30a* is expressed at high level, while *Atpa81, Atp8a2, Atp8b1, Atp8b2, Atp10a, Atp11a* are also expressed in the cerebellum. No ataxia phenotypes were observed in *Atp8a1* and *Atp8b1* KO mice^26^. Given the fact that *Atp8a2* is not expressed in PCs, unidentified PS flippase(s) among *Atp8b2*, *Atp10a*, *Atp11a* may act in PCs together with *Tmem30a*.

## Discussion

In this study, we investigated the role of *Tmem30a* in the PCs of the cerebellum by generating a PC-specific *Tmem30a* KO mouse model. Loss of *Tmem30a* led to early-onset ataxia and PC death starting at P20 (Fig. 2). A detailed analysis revealed increased expression of GFAP and astrogliosis in regions with PC loss (Fig. 4). The elevated expression levels of CHOP and BiP indicated the occurrence of ER stress and unfolded protein response in PCs prior to visible cell loss. Thus, *Tmem30a* deficiency in the PCs resulted in protein folding and transport defects that, in turn, led to apoptosis and gliosis in the cerebellum. Similarly, loss of *Tmem30a* in retinal cone cells has been shown to result in the accumulation of Opsin in the cell body and death of cone cells^40^. An hepatic-specific deletion of *Tmem30a* in the liver caused intrahepatic cholestasis owing to the impaired expression and localization of the bile salt transporters OATP1A4, OATP1B2, NTCP, BSEP, and MRP2^41^. The molecular mechanisms underlying these disease conditions might be similar.

As a β-subunit, TMEM30A is essential for the proper trafficking and functioning of multiple PS flippases, including ATP8B1, ATP11C, ATP8A1, and ATP8A2. *Atp8a1* KO mice exhibit a deficiency in hippocampus-dependent learning^26^. *Atp8a2* mutant mice exhibit ataxia, axonal degeneration, and degeneration of photoreceptor cells and die at 2–3 months of age^24^. Deletion of both *Atp8a2* and *Atp8a1* leads to embryonic lethality^24^. *Tmem30a* KO mice also displayed embryonic lethality, indicating the importance of this gene in vivo^40^. Mutations in *ATP8A2* lead to severe neurological defects characterized by cerebellar atrophy, mental retardation and disequilibrium syndrome in humans^22,23^. Three patients from a large consanguineous family in Turkey were found to harbor a missense mutation of *ATP8A2* (NM_016529 c.1128 C > G, p.I376M)^23^. The proband showed encephalopathy, cerebellar atrophy and mental retardation. Two unrelated patients were reported to exhibit encephalopathy, mental retardation, hypotonia, and optic atrophy. The associated mutations included a homozygous mutation, i.e., c.1287 G > T, p.K429N, and two compound heterozygous mutations, i.e., c.1630G > C, p.A544P and c.1873C > T, p.R625W, in *ATP8A2*. Homozygous mutations affecting the function of *TMEM30A* are likely to cause embryonic lethality. However, genomic changes in the regulatory regions of TMEM30A may lead to disease conditions.

The fact that ATP8A2 is not expressed in PCs, but in deep cerebellar nuclei, indicates other PS flippase as the alpha subunit in PCs (Fig. 7). This result is consistent with our previous study using *Atp8a2* mutant wabbler lethal (*wl*) mice^25^. Here it should be reference 24 *wl* mutant mice displayed neurological phenotypes and exhibited wobble gait, body tremor when walking. Axonal degeneration was prominent in the cerebellum and spinal cord. Central chromatolysis, which indicated axonal injury, was observed in the deep cerebellum nuclei^24^. Disruption of *Atp8a2* in DCN led to degenerated neurons, causing ataxia-like phenotypes). ATP8A2 acts in DCN in the cerebellum. This result provides useful insight for molecular mechanism studies of *ATP8A2-*related cerebellar ataxia, mental retardation, and disequilibrium syndrome^22–25^. Several PS flippases are expressed in the cerebellum (Fig. 7d). Further investigation to identify this unidentified PS flippase and elucidate its in vivo function is necessary to understand the regulation of PS distribution by various flippases and *Tmem30a* in the cerebellum.

Our data revealed the novel roles of *Tmem30a* in PCs in the cerebellum. *Tmem30a* deficiency in PCs resulted in the occurrence of an ER stress response, a substantial decrease in dendritic spine density, significant morphological changes, increased astrogliosis, and PC death. Altogether, our study highlights the importance of *Tmem30a* in cerebellar PCs.

## Electronic supplementary material

Supplementary Data
