# Supplementary material for: Disruption of Tmem30a results in cerebellar ataxia and degeneration of Purkinje cells
Source: Cell Death Dis. 2018 Sep 5;9(9):899. doi: 10.1038/s41419-018-0938-6 (PMC6125289; doi:10.1038/s41419-018-0938-6)
Supplement: Supplementary file 1 — Supplementary Data [file 41419_2018_938_MOESM1_ESM.pdf]

Disruption of *Tmem30a* results in cerebellar ataxia and degeneration of Purkinje cells

Yeming Yang[1], Wenjing Liu[1], Lin Zhang[1], Kuanxiang Sun[1], Kun Peng[1], Shanshan Zhang[1], Shujin Li[3], Mu Yang[3], Zhilin Jiang[1,4], Fang Lu[1], Xianjun Zhu[1-4*]

Supplementary data include supplementary figures Figure S1-8.

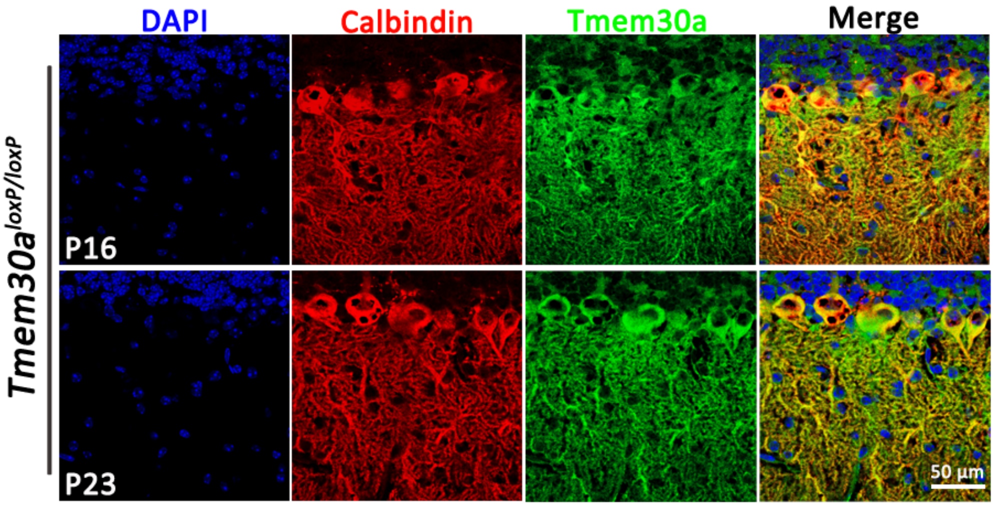

Fig. S1. Expression pattern of *Tmem30a* in the mouse cerebellum. Sagittal sections of cerebellum were immunostained with anti-TMEM30A (green) and Calbindin antibodies (red) and counterstained with DAPI from P16 and P23 WT and KO mice. TMEM30A is expressed in PCs. Scale bar: 50 μm.

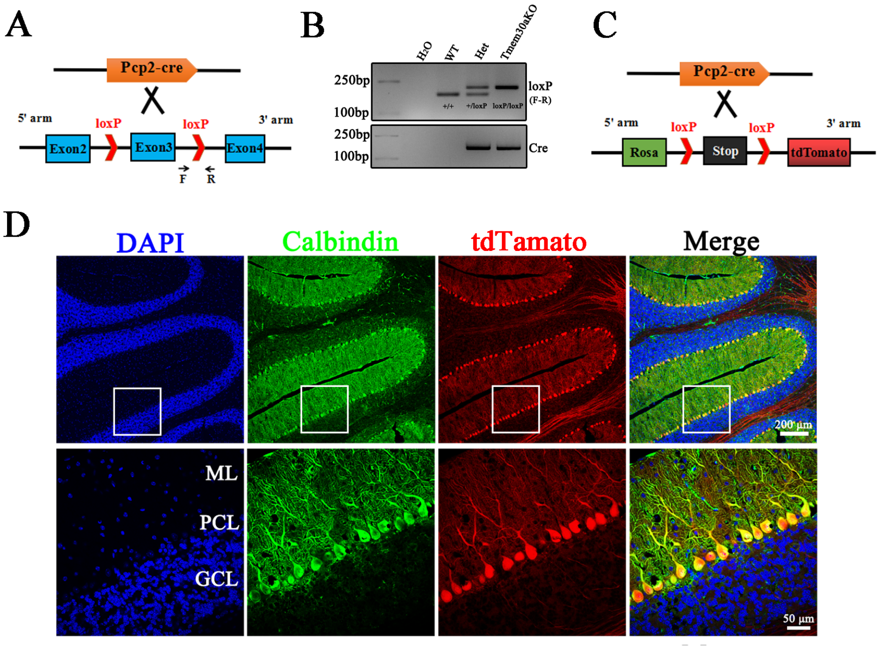

Fig. S2. Generation of *Tmem30a* cerebellar knockout mouse model.

(A) Scheme showing the strategy for generation of *Tmem30a*$^{loxP/loxP}$*; Pcp2-Cre* (named *Tmem30a* KO) mice. In Tmem30a conditional knockout allele, *Exon 3* is flanked by two loxP-sites. Primer pair F-R (showed beneath the diagram) was used to genotype the loxP site downstream of exon 3. When the floxed allele is crossed to Pcp2-cre expressing line, *exon 3* will be deleted specifically in cerebellar Purkinje cells, resulting in a frame-shifting deletion allele and disruption of the *Tmem30a* gene in these cell types. (B) Genotyping of *Tmem30a* KO mice. Genomic DNA from mouse-tail lysate of control (WT), heterozygous (het), and Tmem30a KO mice were amplified using primer pair Tmem30a-loxP-F2 and Tmem30a-loxP-R2. (C) Strategy of generation of tdTomato reporter line by crossing Pcp2-Cre transgene line with tdTomato reporter allele. TdTomato reporter was knocked into the Rosa26 locus and driven by the universal promoters. A stop codon, flanked by two loxP sites, was placed upstream of the reporter cassettes, preventing reporter expression in the absence of Cre-mediated recombination. When Pcp2-Cre is present, Pcp2-Cre mediated recombination removes the stop codon, allowing the expression of the red fluorescent tdTomato reporter. (D) A ROSA-tdTomato reporter was introduced into the mice to monitor Cre activity. PCs are labeled with Calbindin antibody. Nuclei were counter-stained with DAPI (blue). The tdTomato expressing cells were distinctly immunoreactive for Calbindin in the cerebellum, indicated that Pcp2-cre is specifically expressed in PCs.

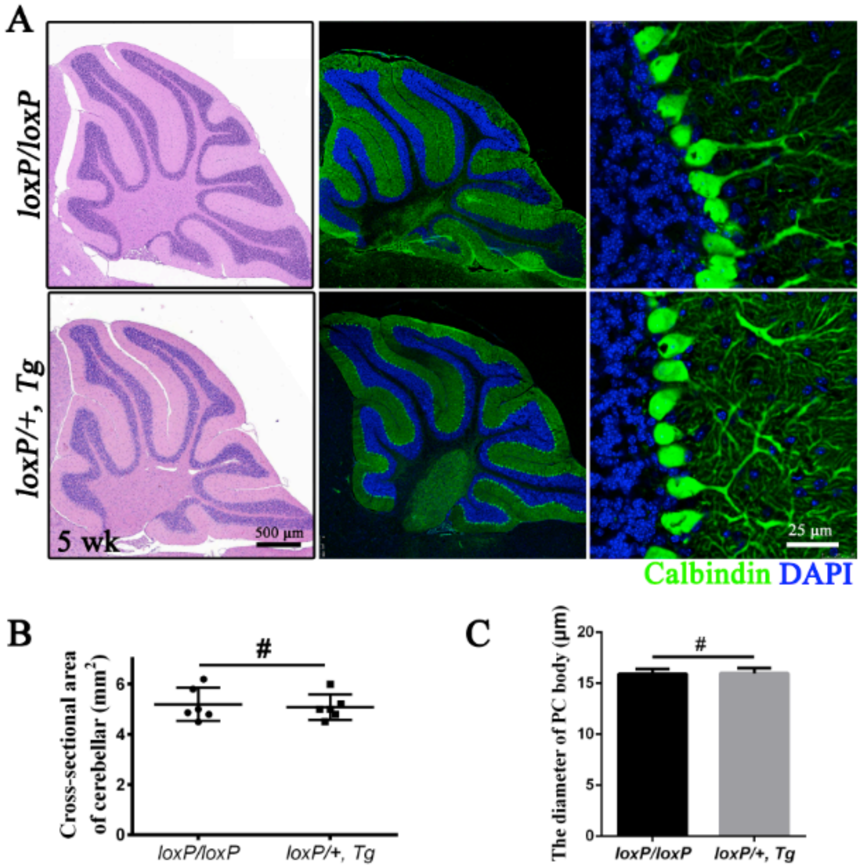

Fig. S3. The *Tmem30a* heterozygous cerebellum appeared to be structurally normal compared to their control littermates. (A) Histological detection of celbellum from WT (*loxP/loxP*) and heterozygous (*loxP/loxP, Tg*) mice at the age of 5 week. Left panel presents the images of H&E staining; middel panel reflects Immunofluorescence labeling of cerebellum cryosections with calbindin (green) and DAPI (blue). Higher magnification images of representative calbindin positive staining PCs (arrowheads) were shown in the right panel. (B) Quantification of the cross-sectional area of cerebellum (n=3). (C) The diameter of PC body of 5 week old heterozygous and control mice (n=3). The data represent means±SEM.

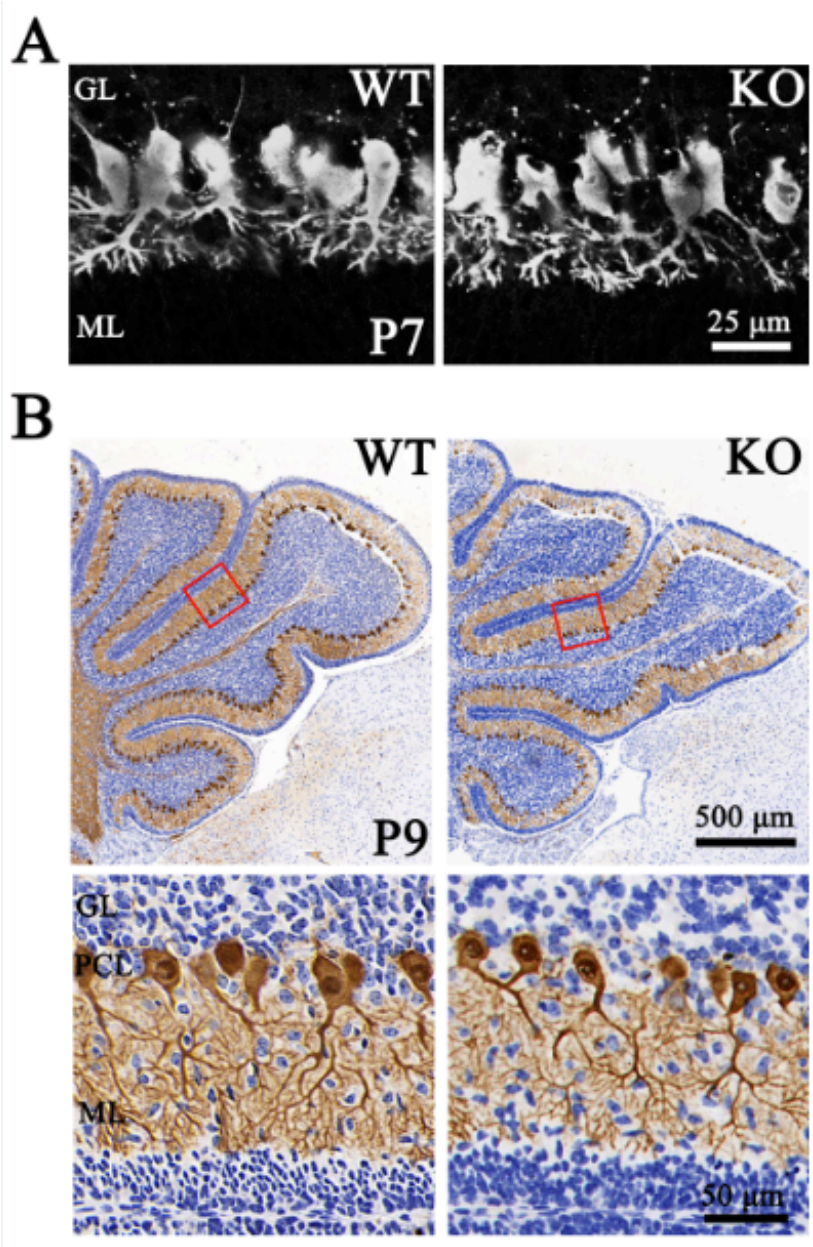

Fig. S4. The structural changes of PCs at early stages of development in *Tmem30a* KO cerebellum. (A) Raw images of Calbindin positive staining PCs in cerebellum from P7 WT and KO mice. No significant difference was observed. (B) Sagittal sections of cerebella immunostained with Calbindin antibodies (brown) and counterstained with hematoxylin from P9 WT and KO mice. The lower panel exhibited the higher-magnification images of boxed areas in the top panel. Calbindin staining revealed loss of dendrites in *Tmem30a* KO Purkinje cells compared with WT.

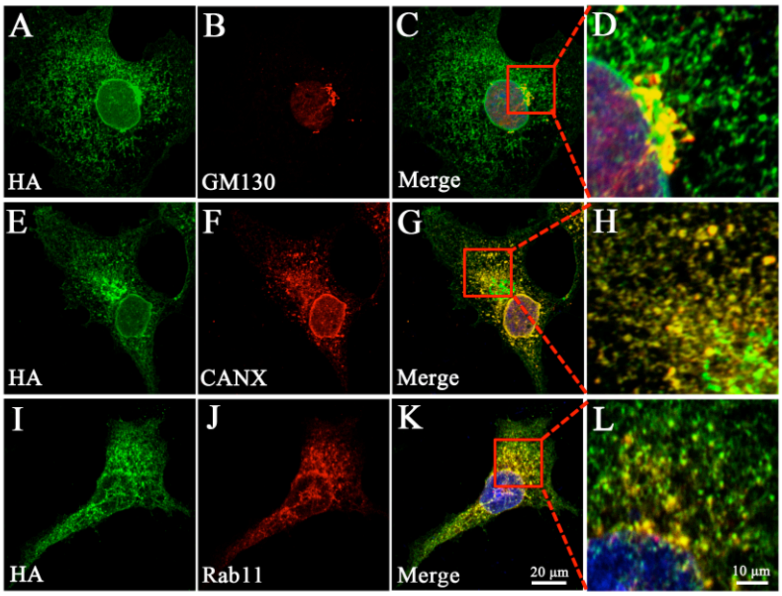

Fig. S5. TMEM30A is located in ER, trans-Glogi network (TGN) and early endosome in COS7 cells.

COS7 cells were transiently transfected with an expression vector for HA-tagged TMEM30A. Transfected cells were stained with anti-HA antibody (green), and the same specimens were co-stained with the TGN marker GM130 (A-C), the ER marker CANX (E-G) and the early endosome marker Rab11a (I-K) respectively (red). Right panel (D, H, L) show the higher-magnification images of boxed areas.

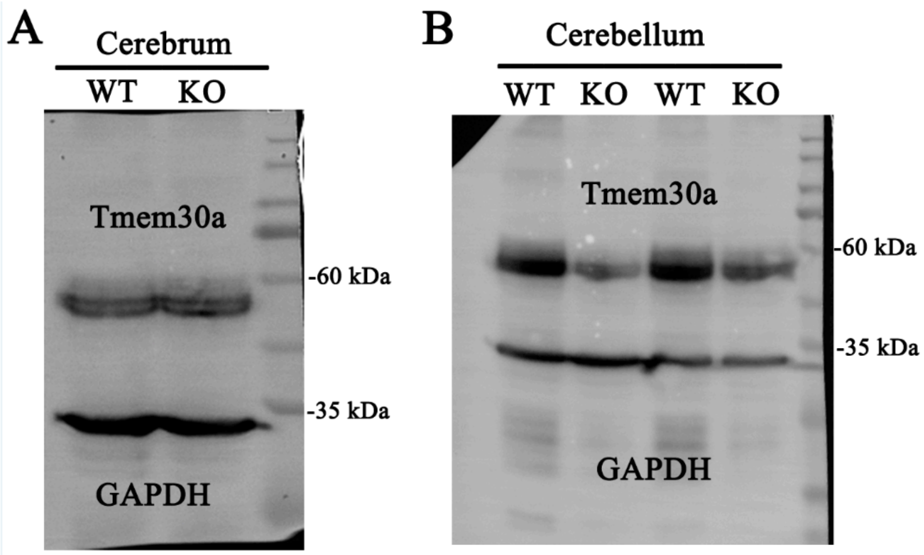

Fig. S6. Uncropped Immunoblotting images of total lysates of the cerebrum and cerebellum from WT and KO mice, probed with TMEM30A antibodies.

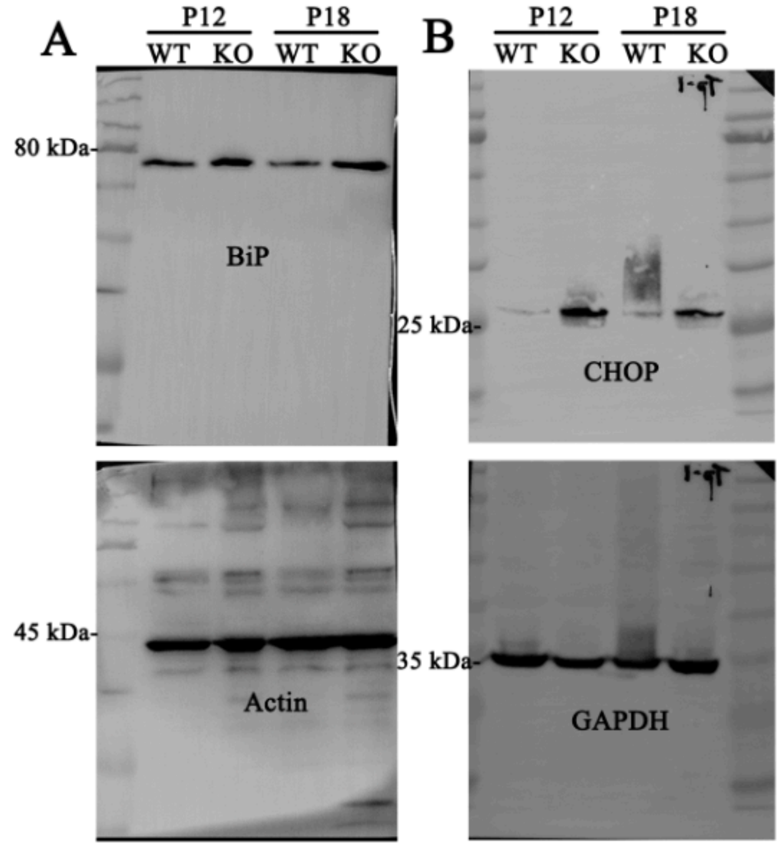

Fig. S7. Uncropped Immunoblotting images of cerebellar protein extracts from WT and KO mice respectively, probed with antibodies against ER stress markers BiP and CHOP.

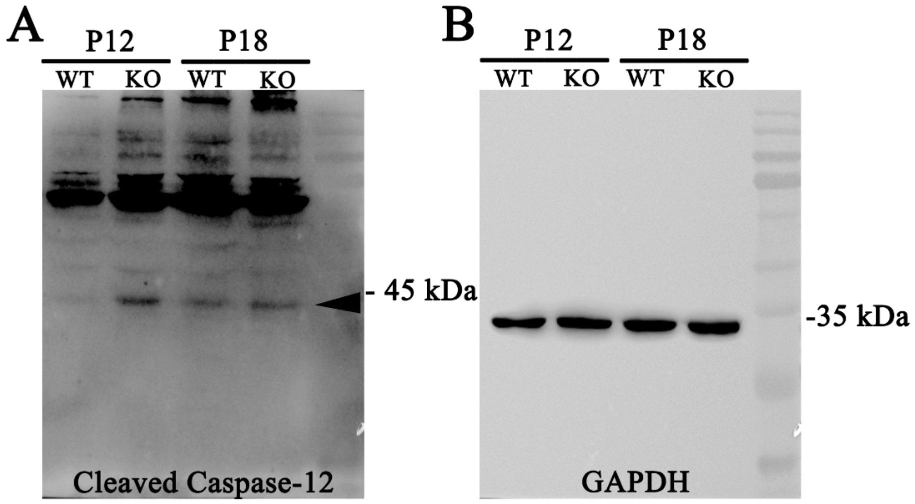

Fig. S8. Uncropped Immunoblotting images of cerebellar protein extracts from WT and KO mice at P16 and P20 respectively, probed with antibodies against apoptosis marker cleaved caspase-3.
